# Mycosis in Wounds

**Published:** 1916-03

**Authors:** 


**Mycosis in Wounds.** E. Rouyer and J. Pellissier, Acad. de Med., 1915, page 307, have isolated by cultures on sterile carrots, white tufts, identified as saccharomyces tumifaciens. Cospora has also been found. Iodine, peroxid of hydrogen, are contraindicated. Copper sulphate is advised.

